# Human cultured dendritic cells show differential sensitivity to chemotherapy agents as assessed by the MTS assay

**DOI:** 10.1038/sj.bjc.6694366

**Published:** 1999-12

**Authors:** D Chao, P Bahl, S Houlbrook, L Hoy, A L Harris, J M Austyn

**Affiliations:** Nuffield Department of Surgery, John Radcliffe Hospital, Headington, Oxford OX3 9DU, UK; ICRF Medical Oncology Unit, The Churchill Hospital, Headington, Oxford OX3 7LJ, UK

**Keywords:** human, dendritic cell, chemotherapy, MTS, immunotherapy

## Abstract

Assessment of the chemosensitivity of dendritic cells (DC) may allow more rational development of combined chemotherapy and immunotherapy protocols. Human monocyte-derived DC generated reproducible results in the MTS (Owen’s reagent) assay, which was then used to study DC survival after treatment with four different chemotherapy agents. DC preparations from three different donors were used per drug. DC were sensitive to doxorubicin (concentration range 0.1–50 μM) with variation in sensitivity between donors (IC_50_ 244–1100 nM). The most extreme variation was seen for vinblastine (concentration range 250–0.025 μM with IC_50_ 0.15–17.25 μM). In contrast, there was relative resistance to etoposide (concentration range 0.2–200 μM) and 5-fluorouracil (concentration range 0.7–7700 μM) with no toxicity seen until 50 μM and 770 μM respectively. The function of DC in allogeneic mixed leucocyte reactions closely paralleled results from the MTS assays. The differential sensitivity to chemotherapy agents did not appear to be due to expression of P-glycoprotein. These results suggest that etoposide or 5-fluorouracil is less likely to reduce the immunotherapeutic potential of DC and may be valuable in the design of prodrug activation therapy. © 1999 Cancer Research Campaign


*British Journal of Cancer* (1999) **81**(8), 1280–1284
© 1999 Cancer Research Campaign
Article no. bjoc.1999.0842

### Human cultured dendritic cells show differential sensitivity to chemotherapy agents as assessed by the MTS assay

**D Chao[1], P Bahl[1], S Houlbrook[2], L Hoy[1], AL Harris[2] and JM Austyn[1]**

[1]Nuffield Department of Surgery, John Radcliffe Hospital, Headington, Oxford OX3 9DU, UK; [2]ICRF Medical Oncology Unit, The Churchill Hospital, Headington, Oxford OX3 7LJ, UK

**Summary** Assessment of the chemosensitivity of dendritic cells (DC) may allow more rational development of combined chemotherapy and immunotherapy protocols. Human monocyte-derived DC generated reproducible results in the MTS (Owen's reagent) assay, which was then used to study DC survival after treatment with four different chemotherapy agents. DC preparations from three different donors were used per drug. DC were sensitive to doxorubicin (concentration range 0.1–50 μM) with variation in sensitivity between donors ($IC_{50}$ 244–1100 nM). The most extreme variation was seen for vinblastine (concentration range 250–0.025 μM with $IC_{50}$ 0.15–17.25 μM). In contrast, there was relative resistance to etoposide (concentration range 0.2–200 μM) and 5-fluorouracil (concentration range 0.7–7700 μM) with no toxicity seen until 50 μM and 770 μM respectively. The function of DC in allogeneic mixed leucocyte reactions closely paralleled results from the MTS assays. The differential sensitivity to chemotherapy agents did not appear to be due to expression of P-glycoprotein. These results suggest that etoposide or 5-fluorouracil is less likely to reduce the immunotherapeutic potential of DC and may be valuable in the design of prodrug activation therapy. © 1999 Cancer Research Campaign

**Keywords:** human; dendritic cell; chemotherapy; MTS; immunotherapy

Dendritic cells (DC), as the most potent antigen-presenting cells known, are of increasing interest in tumour immunotherapy (Austyn, 1998). There is evidence that gene therapy, using both prodrug activation and cytokines, acts by stimulating immune responses to dying tumour cells (Melcher et al, 1998) and that DC may be important in initiating these responses, perhaps by the uptake of apoptotic tumour cells and the presentation of tumour antigens (Rubartelli et al, 1997*b*; Albert et al, 1998). DC generated in vitro from monocytes using granulocyte–macrophage colony-stimulating factor (GM-CSF) and interleukin-4 (IL-4) are being used widely in clinical trials to assess directly DC efficacy in inducing immune responses against tumours (Bender et al, 1996; Nestle et al, 1998). Combining chemotherapy and immunotherapy is a logical extension of current trials and is already in use for some tumours, such as renal carcinoma and melanoma. The chemosensitivity of DC is unknown but is important to the rational development of combined modality regimens in which chemotherapy agents do not detract from the immune response against the tumour. In this paper we used the MTS (Owen's reagent) assay to study the effects of several different classes of cytotoxic drugs on DC survival and demonstrated a marked difference in sensitivity both to different classes of chemotherapy agents and between individuals.

*Received 17 February 1999*
*Revised 4 June 1999*
*Accepted 8 June 1999*

*Correspondence to:* D Chao

### MATERIALS AND METHODS

### Generation of cultured DC

Commercial buffy coats were obtained from Bristol Blood Transfusion Service (Bristol, UK) and were all from normal blood donors. Peripheral blood mononuclear cells were isolated using Lymphoprep (Nycomed, Norway) as per manufacturer's instructions. Human monocyte-derived DC were generated as described by Bender et al (1996) up to day 6 to obtain immature DC and were not matured further. Culture medium was RPMl-1640 (Life Technologies, Paisley, UK) supplemented with 1% autologous plasma, 50 ng $ml^{-1}$ GM-CSF (Novartis, Camberley, UK) and 50 ng $ml^{-1}$ IL-4 (Peprotech, Boston, MA, USA). Contaminating lymphocytes were removed with anti-CD3 and anti-CD19 antibodies (Dako, Glostrup, Denmark) followed by M-450 Dynabeads coated with sheep anti-mouse antibody (Dynal, Oslo, Norway) as per manufacturer's instructions. The resultant preparation routinely contained > 90% DC as defined by high expression of HLA-DR and CD86 and lack of expression of CD3, CD19 and CD14 by flow cytometry (data not shown).

### MTS assay

Purified DC were plated in flat-bottomed 96-well plates at $10^5$, $5 \times 10^4$, $10^4$, $5 \times 10^3$ and $10^3$ DC per well in triplicate to generate standard curves. To assess DC survival after chemotherapy, $5 \times 10^4$ DC were plated per well in triplicate for each concentration of cytotoxic drug. Doxorubicin (Farmitalia, St Albans, UK), an anthracycline and topoisomerase II inhibitor which also generates

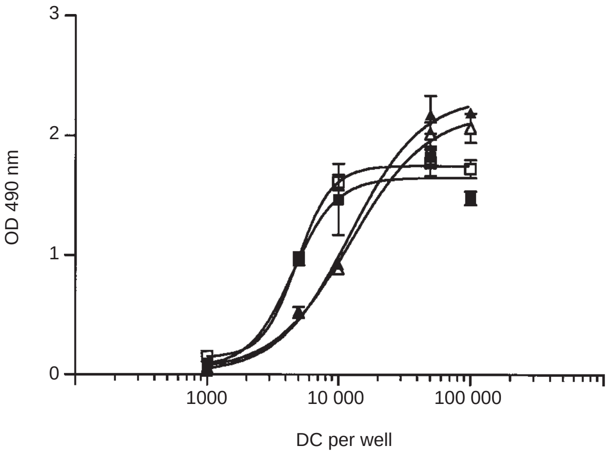

**Figure 1** Standard curves generated by DC in the MTS assay from two different donors in duplicate experiments at different times. Donor A, experiment 1 (□) and experiment 2 (■). Donor B, experiment 1 (▲) and experiment 2 (△). Bars, s.d.

free radicals, was used at 0.1, 1, 5, 10 and 50 μM. Etoposide (Bristol-Myers Squibb, Hounslow, UK), a podophyllotoxin and topoisomerase II inhibitor without free radical generation, was used at 0.2, 2, 20, 100 and 200 μM. 5-Fluorouracil (5-FU; Roche, Welwyn Garden, UK), an antimetabolite, was used at 0.7, 7, 77, 770 and 7700 μM (corresponding to 0.1, 1, 10, 100 and 1000 μg $ml^{-1}$). Vinblastine (Eli Lilly, Basingstoke, UK), a vinca alkaloid, was used (at 0.025, 0.25, 2.5, 25 and 250 μM). Drugs were diluted in complete medium immediately before addition to DC cultures. DC were incubated with drugs for 2 h at 37°C, 5% carbon dioxide, washed twice with fresh medium and incubated for a further 4 days before MTS assay (Promega, Madison, WI, USA). Results for standard curves are expressed as the mean optical density (OD) of triplicate wells ± standard deviation (s.d.) to illustrate differences in colorimetric change seen between donors, or as percentage survival of DC ± s.d. to obtain the $IC_{50}$ which is the dose of drug causing 50% reduction in DC survival. To validate the MTS assay against conventional cell counting, one set of experiments for each chemotherapy agent included a parallel 96-well plate, which was assessed by counting five fields of view at ×200 magnification per well following trypan blue. All cell counts were carried out by the same individual who was blinded to the MTS assay results for that experiment. The curves were generated by non-linear regression fit using the Prism program (GraphPad Software, San Diego, CA, USA). Each cytotoxic drug was assayed in duplicate with three different donors.

### Allogeneic mixed lymphocyte reaction

DC were exposed to chemotherapy agents as described above. Varying numbers of DC were then cultured with a constant number of $10^5$ nylon wool purified allogeneic T-cells (highest concentration $10^4$ DC per well with successive halving dilutions) for 5 days (Romani et al, 1996). T-cell proliferation was measured by [$^3$H]thymidine incorporation (0.5 μCi $well^{-1}$) and expressed as the mean cpm of triplicate wells ± s.d.

### Assays for P-glycoprotein expression

DC were stained for P-glycoprotein (P-gp) expression using monoclonal antibody UIC-2 (Immunotech, Marseille, France) and

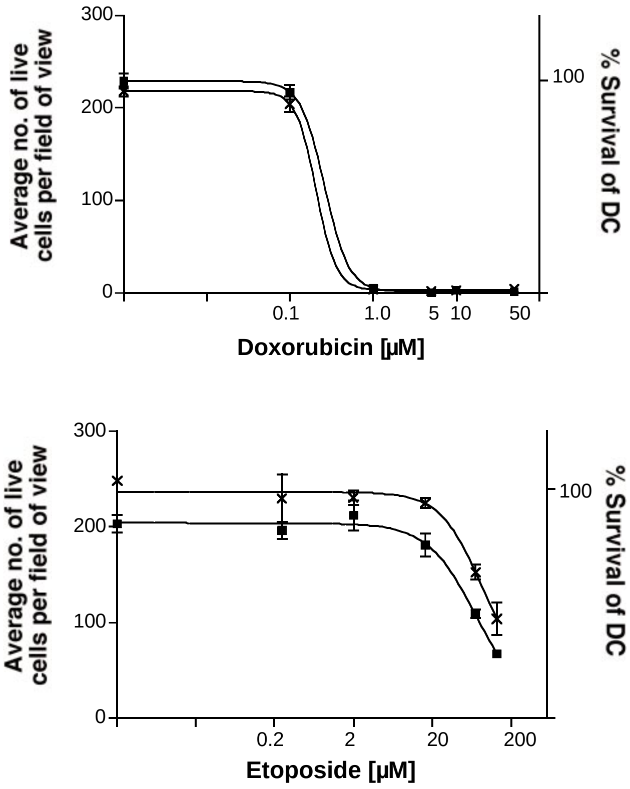

**Figure 2** (**A**) Sensitivity of DC from donor C to doxorubicin as assessed by the MTS assay (■) or by cell counting (x). The $IC_{50}$ given by the MTS assay is 207 nM and by cell counting is 262 nM. Bars, s.d. (**B**) Sensitivity of DC from donor C to etoposide as assessed by the MTS assay (■) or by cell counting (x). The $IC_{50}$ given by the MTS assay is 120 μM and by cell counting is 102 μM. Bars, s.d.

rabbit anti-mouse $F(ab)_2$– fluorescein isothiocyanate (FITC; Dako, Glostrup, Denmark) for flow cytometry as per manufacturers' instructions. For the functional assay, MTS plates were set up as above using a donor who had previously exhibited high resistance to vinblastine. One set of DC were treated with vinblastine as previously, and a parallel set was treated with vinblastine and verapamil (20 μM, Abbott Laboratories, Maidenhead, UK) simultaneously. The breast cancer cell line MC7F-Adr, which expresses high levels of P-gp, was used as the positive control for assays.

### RESULTS

### Human dendritic cells generated reproducible results in the MTS assay

The MTS assay provided a reliable technique to count DC. Figure 1 demonstrates three points. First, DC were capable of generating significant colorimetric change in the MTS assay and the $OD_{490}$ correlated with numbers of DC present over a wide range ($10^3$ to greater than $10^5$). Lymphocytes did not generate a significant colorimetric change and, even though we routinely purified our DC populations, parallel experiments with non-purified DC showed that contaminating lymphocytes did not contribute significantly to the colourimetric change (data not shown). Second, DC gave reproducible results. Standard curves from separate donors were different but were reproducible on different DC preparations

*© 1999 Cancer Research Campaign*

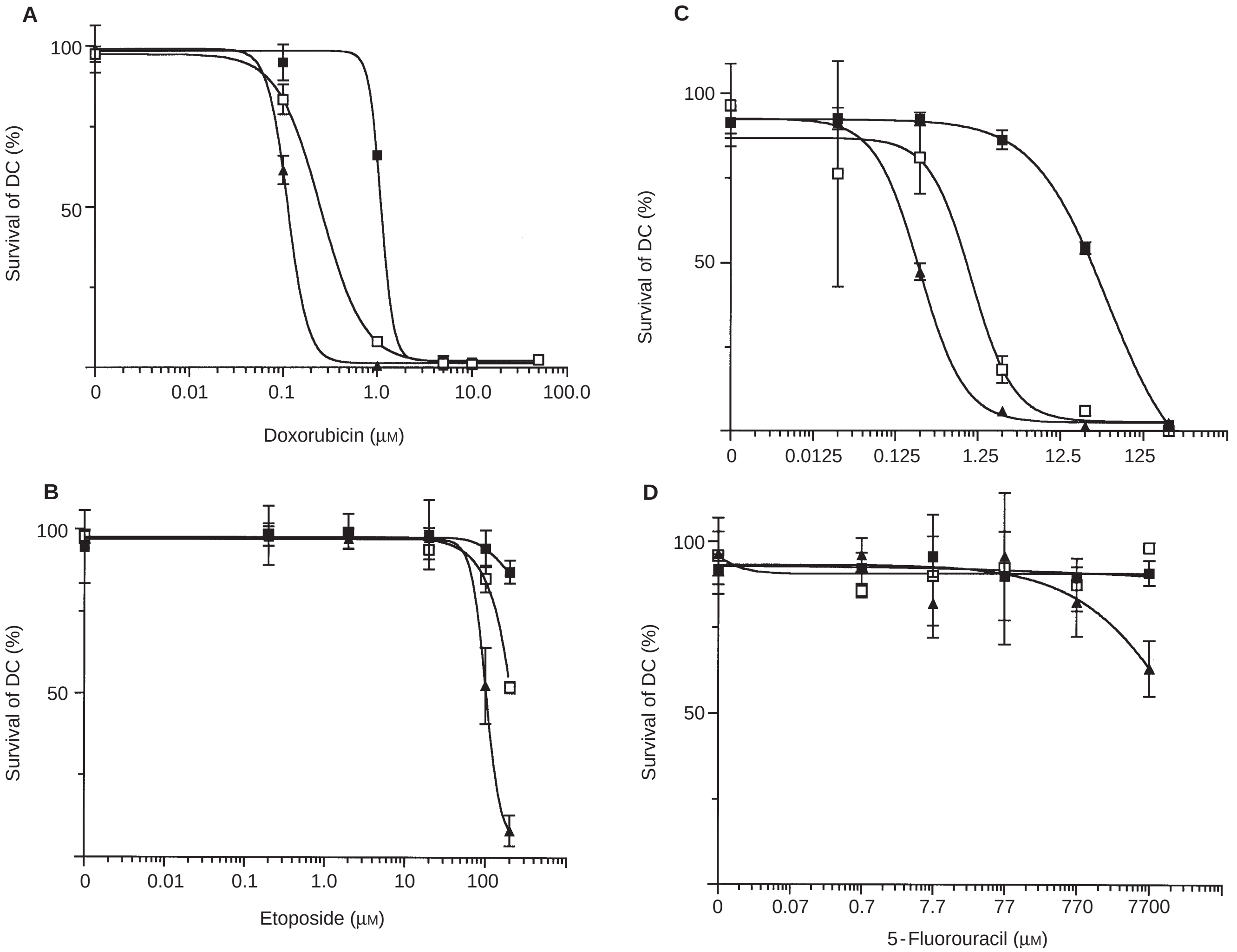

**Figure 3** (**A**) Sensitivity of DC to doxorubicin in the MTS assay. Donor D (■), donor E (□) and donor F (▲). Bars, s.d. (**B**) Sensitivity of DC to etoposide in the MTS assay. Same three donors as shown in **A**. Donor D (■), donor E (□) and donor F (▲). Bars, s.d. (**C**) Sensitivity of DC to vinblastine in the MTS assay. Same three donors as shown in **A**. Donor D (■), donor E (□) and donor F (▲). Bars, s.d. (**D**) Sensitivity of DC to 5-fluorouracil in the MTS assay. Donor G (▲), donor H (□) and donor K (■). Bars, s.d.

assayed at different times. Third, the standard curves showed that an apparent plateau value of colorimetric change occurred at around $5 \times 10^4$ cells well$^{-1}$, and therefore this cell concentration was selected for all subsequent survival experiments with chemotherapy agents.

### MTS assay results correlated with conventional cell counting

It was important to confirm that the MTS assay gave comparable results to conventional cell counting and to exclude the possibility that the chemotherapy agents interfered in any way with the MTS assay to give false results. Figure 2 shows the results of two parallel plates of DC from one donor treated with doxorubicin or etoposide and read by either the MTS assay or conventional cell counting. The $IC_{50}$ for doxorubicin and etoposide for this donor are highly similar whether derived by the MTS assay or conventional cell counting.

### DC were sensitive to doxorubicin and vinblastine but resistant to etoposide and 5-FU

Figure 3A shows that DC were sensitive to doxorubicin and that DC from different individuals showed varying sensitivities with $IC_{50}$ of 244 nM (▲), 253 nM (□) and 1100 nM (■). In contrast, DC from the same three donors showed relative resistance to the effect of etoposide (Figure 3B) with no toxicity evident until 50 μM for all donors. We also tested longer exposures to etoposide, up to 24 h, with no observed differences (data not shown). The greatest variation in sensitivity was seen for vinblastine (Figure 3C) with $IC_{50}$ of 0.15 μM (▲), 1 μM (□) and 17.25 μM (■), which is three log orders difference in sensitivity for these three donors. DC were resistant to 5-FU (Figure 3D), with the $IC_{50}$ not being reached for any donor and there was no significant toxicity up to 770 μM.

### DC function in the allogeneic MLR correlated with the MTS assay

DC from donor D were relatively insensitive to etoposide at the highest concentration of 200 μM and this was reflected in the

*© 1999 Cancer Research Campaign*

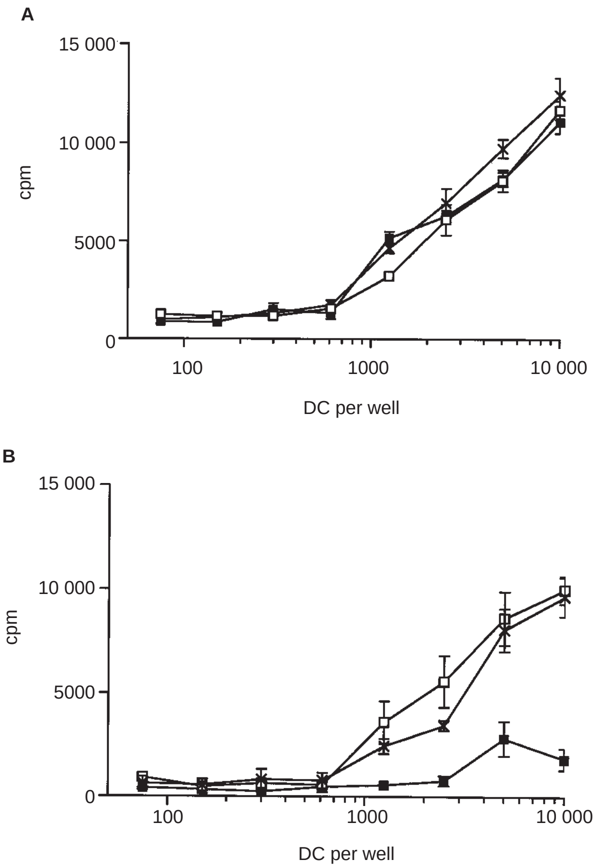

**Figure 4** (**A**) Function of DC from donor D in allogeneic MLR after treatment with etoposide. Untreated DC (X), DC treated with 200 nM etoposide (□) and DC treated with 200 μM etoposide (■). Bars, s.d. (**B**) Function of DC from Donor F in allogeneic MLR after treatment with etoposide. Untreated DC (X), DC treated with 200 nM etoposide (□) and DC treated with 200 μM etoposide (■). Bars, s.d.

allogeneic MLR (Figure 4A) with normal stimulatory activity up to 200 μM. In contrast, DC from donor F was sensitive to etoposide at the highest concentration of 200 μM, and this correlated with significantly reduced activity at 200 μM (Figure 4B). Similar data were obtained following exposure to the other drugs (data not shown).

### Differential sensitivity to chemotherapy agents was not due to expression of P-gp

There was no expression of P-gp on DC by flow cytometry (data not shown). In case P-gp was expressed at levels below the threshold of detection by flow cytometry, we used a functional inhibition assay using verapamil at a concentration previously shown to be inhibitory for P-gp (Randolph et al, 1998). There was no increase in the chemosensitivity to vinblastine, and the similarly treated standards showed that the verapamil had not affected the MTS assay (data not shown).

### DISCUSSION

The MTS assay depends upon mitochondrial function and can be used as a measure of cell viability. It is routinely used to assess the chemosensitivity of tumour cell lines by inhibition of proliferation (Cory et al, 1991). However, monocyte-derived DC are non-proliferating cells and we adapted the MTS assay to measure survival of DC after exposure to chemotherapy agents. We have shown that the MTS assay gives reproducible results which are consistent with DC survival assessed by conventional cell counting with trypan blue exclusion. The major advantage of the MTS assay is that it is automated and therefore much simpler than manual cell counting. We chose four different cytotoxic drugs which are representative of chemotherapy agents commonly used for the treatment of cancers in which immunotherapy is also being investigated, and at doses which pharmacokinetic studies suggest are achievable in vivo.

The results for doxorubicin and etoposide are considered together because the same three donors were used and both drugs act on topoisomerase II. Etoposide is more specific and only inhibits proliferating cells, which may account for its low toxicity for non-proliferating DC. In contrast, doxorubicin has multiple sites of action including free radical generation. A bolus of doxorubicin, 75 mg $m^{-2}$, gave a peak plasma concentration of 5 μM falling off to 0.1 μM by 1 h, followed by a long half-life of 30 h (Greene et al, 1983); these concentrations were toxic to DC in this study. A bolus of etoposide, 125 mg $m^{-2}$, gave a peak plasma concentration of 40 μM, with clearance by 24 h (Henwood and Brogden, 1990). We have shown that there is no toxicity of this drug in the MTS assay up to 50 μM for 2 and 24 h.

Pharmacokinetic studies for vinblastine after a bolus of 10 mg showed a peak plasma concentration of 0.25 μM falling to 1 nM after 2 h (Nelson et al, 1980). The variation in sensitivity for vinblastine was so large that at these plasma concentrations DC from some individuals may be sensitive, whereas others may be completely resistant. Vinblastine inhibits the polymerization of tubulin and the formation of the mitotic spindle. Thus resistance may be related to non-proliferation of DC, but the variable toxicity suggests that there may be other factors.

The same three donors (donors D, E and F) showed variable resistance to doxorubicin and vinblastine but all showed relative resistance to etoposide. This pattern of chemoresistance has similarities to the classic multidrug resistance conferred by P-gp, the product of the MDR-1 gene. P-gp has recently been identified on Langerhans cells and may have a role in cell migration (Randolph et al, 1998). We were unable to demonstrate the expression of P-gp on monocyte-derived DC by flow cytometry or to show a functional effect on vinblastine sensitivity by verapamil inhibition. The multidrug resistance-associated protein (MRP) is unlikely to be involved because MRP overexpression is associated with resistance to etoposide but not to vinblastine (Berger et al, 1997).

Pharmacokinetic studies for 5-FU after a bolus of 900 mg $m^{-2}$ showed peak plasma concentration of 300 μM with undetectable levels by 2 h (Fraile et al, 1980). In the MTS assay no toxicity is seen up to 770 μM for 2 h, suggesting that bolus administration of 5-FU will not be toxic to DC. 5-FU is incorporated into both RNA and DNA and the basis of DC resistance is not clear.

It is possible that chemotherapy agents may alter DC function without affecting viability. To address this issue we assessed DC stimulatory function with the allogeneic MLR which is a standard assay. We acknowledge that the MLR does not assess all aspects of DC function, but it is functionally relevant as it correlates well with other parameters of DC stimulatory function such as expression of costimulatory molecules (Bender et al, 1996), and currently many mechanisms of DC function are poorly understood and so cannot be assessed. These limitations add to the difficulties

© 1999 Cancer Research Campaign of extrapolating from the in vitro model to the in vivo situation, but the data presented here at least allows us to formulate clinical models which might be tested. Thus the low toxicity of etoposide and 5-FU to DC at clinically relevant plasma levels suggest their use in combined modality studies. 5-FU is of interest because it is already in use in combined modality treatment with IL-2 and interferon-α in renal carcinoma, and 5-FU can be given as a prodrug, 5-fluorocytosine, in gene therapy (Mullen et al, 1992). Recent studies suggest that DC present antigens acquired by uptake of apoptotic cells (Rubartelli et al, 1997*a*; Albert et al, 1998) and we are investigating whether 5-FU and etoposide might enhance DC immunotherapy through tumour apoptosis without causing toxicity to DC.

### REFERENCES

Albert ML, Sauter B and Bhardwaj N (1998) Dendritic cells acquire antigen from apoptotic cells and induce class I-restricted CTL. *Nature* **392**: 86–89

Austyn JM (1998) Dendritic cells. *Curr Opin Hematol* **5**: 3–15

Bender A, Sapp M, Schuler G, Steinman RM and Bhardwaj N (1996) Improved methods for the generation of dendritic cells from non-proliferating progenitors in human blood. *J Immunol Methods* **196**: 121–135

Berger W, Elbling L, Hauptmann E and Micksche M (1997) Expression of the multidrug resistance-associated protein (MRP) and chemoresistance of human non-small cell lung cancer cells. *Int J Cancer* **73**: 84–93

Cory AH, Owen TC, Barltrop JA and Cory JG (1991) Use of an aqueous soluble tetrazolium/formazan assay for cell growth assays in culture. *Cancer Commun* **3**: 207–212

Fraile RJ, Baker LH, Buroker TR, Horwitz J and Vaitkevicius VK (1980) Pharmacokinetics of 5-fluorouracil administered orally, by rapid intravenous and by slow infusion. *Cancer Res* **40**: 2223–2228

Greene RF, Collins JM, Jenkins JF, Speyer JL and Myers CE (1983) Plasma pharmacokinetics of Adriamycin and Adriamycinol: implications for the design of in vitro experiments and treatment protocols. *Cancer Res* **43**: 3417–3421

Henwood JM and Brogden RN (1990) Etoposide: a review of its pharmacodynamic and pharmacokinetic properties, and therapeutic potential in combination chemotherapy of cancer. *Drugs* **39**: 438–490

Melcher A, Todryk S, Hardwick N, Ford M, Michael J and Vile RG (1998) Tumour immunogenicity is determined by the mechanism of cell death via induction of heat shock protein expression. *Nat Med* **4**: 581–587

Mullen CA, Kilstrup M and Blaese RM (1992) Transfer of the bacterial gene for cytosine deaminase to mammalian cells confers lethal sensitivity to 5-fluorocytosine: a negative selection system. *Proc Natl Acad Sci* **89**: 33–37

Nelson RI, Dyke RW and Root MA (1980) Comparative pharmacokinetics of vindesine, vincristine and vinblastine in patients with cancer. *Cancer Treat Rev* **7**: 17–24

Nestle FO, Alijagic S, Gilliet M, Sun Y, Grabbe S, Dummer R, Burg G and Schadendorf D (1998) Vaccination of melanoma patients with peptide- or tumour lysate-pulsed dendritic cells. *Nat Med* **4**: 328–335

Randolph GJ, Beaulieu S, Pope M, Sugawara I, Hoffman L, Steinman RM and Muller WA (1998) A physiological function for p-glycoprotein during the migration of dendritic cells from skin via afferent lymphatic vessels. *Proc Natl Acad Sci USA* **95**: 6942–6929

Romani N, Reider D, Heuer M, Ebner S, Kampgen E, Eibl B, Niederwieser D and Schuler G (1996) Generation of mature dendritic cells from human blood. An improved method with special regard to clinical applicability. *J Immunol Methods* **196**: 137–151

Rubartelli A, Poggi A and Zocchi MR (1997*a*). The selective engulfment of apoptotic bodies by dendritic cells is mediated by the αβ3 integrin and requires intracellular and extracellular calcium. *Eur J Immunol* **27**: 1893–1900

Rubartelli A, Poggi A and Zocchi MR (1997*b*). The selective engulfment of apoptotic bodies by dendritic cells is mediated by the alpha(v)beta3 integrin and requires intracellular and extracellular calcium. *Eur J Immunol* **27**: 1893–1900.

---

Dr David Chao is a Medical Research Council Clinical Training Fellow.

*© 1999 Cancer Research Campaign*
